# Inhibiting Basal Ganglia Regions Reduces Syllable Sequencing Errors in Parkinson's Disease: A Computer Simulation Study

**DOI:** 10.3389/fncom.2018.00041

**Published:** 2018-06-06

**Authors:** Valentin Senft, Terrence C. Stewart, Trevor Bekolay, Chris Eliasmith, Bernd J. Kröger

**Affiliations:** ^1^Medical School, RWTH Aachen University, Aachen, Germany; ^2^Centre for Theoretical Neuroscience, University of Waterloo, Waterloo, ON, Canada; ^3^Applied Brain Research, Waterloo, ON, Canada; ^4^Department for Phoniatrics, Pedaudiology, and Communication Disorders, RWTH Aachen University, Aachen, Germany

**Keywords:** deep brain stimulation, basal ganglia, neural engineering framework, Parkinson's disease, speech, speech disorders

## Abstract

**Background:** Parkinson's disease affects many motor processes including speech. Besides drug treatment, deep brain stimulation (DBS) in the subthalamic nucleus (STN) and globus pallidus internus (GPi) has developed as an effective therapy.

**Goal:** We present a neural model that simulates a syllable repetition task and evaluate its performance when varying the level of dopamine in the striatum, and the level of activity reduction in the STN or GPi.

**Method:** The Neural Engineering Framework (NEF) is used to build a model of syllable sequencing through a cortico-basal ganglia-thalamus-cortex circuit. The model is able to simulate a failing substantia nigra pars compacta (SNc), as occurs in Parkinson's patients. We simulate syllable sequencing parameterized by (i) the tonic dopamine level in the striatum and (ii) average neural activity in STN or GPi.

**Results:** With decreased dopamine levels, the model produces syllable sequencing errors in the form of skipping and swapping syllables, repeating the same syllable, breaking and restarting in the middle of a sequence, and cessation (“freezing”) of sequences. We also find that reducing (inhibiting) activity in either STN or GPi reduces the occurrence of syllable sequencing errors.

**Conclusion:** The model predicts that inhibiting activity in STN or GPi can reduce syllable sequencing errors in Parkinson's patients. Since DBS also reduces syllable sequencing errors in Parkinson's patients, we therefore suggest that STN or GPi inhibition is one mechanism through which DBS reduces syllable sequencing errors in Parkinson's patients.

## Introduction

Parkinson's disease has a complex pathology and there are open questions regarding the genesis of the illness. The main mechanism seems to be the degradation of the substantia nigra in the basal ganglia (BG), which results in reduced levels of the neurotransmitter dopamine in the striatum (Goetz and Pal, 2014). Symptoms caused by this disease include bradykinesia, akinesia, tremors, rigor, postural instability, freezing of gait, and freezing of speech (Erro et al., 2014; Vercruysse et al., 2014). Syllable repetition tasks, also called diadochokinesia tasks, are often used to identify problems in speech production related to Parkinson's disease (Ackermann et al., 1993).

The BG can be understood as a complex system of excitation, inhibition and disinhibition. Together with the thalamus (Thal), the BG initiates actions like the production of the next syllable during speaking, or making the next step during walking. Candidate actions are first activated at cortical levels and subsequently execute upon receiving a go-signal from the BG-Thal-system. Here, we focus on the role of the BG as an action selection system. Gurney et al. (2001a,b) describe action selection in the BG as involving two separate pathways, the action selection and the control pathways. A signal representing an action coming from the cortex to the BG first gets processed by the striatum and the STN. In the selection pathway, the substantia nigra pars compacta (SNc) excites the striatum by through D1 dopamine receptors. In the control pathway, the substantia nigra pars compacta (SNc) inhibits the striatum through D2 dopamine receptors. The striatum inhibits other nuclei with the neurotransmitter GABA. The activation of D1 receptors in the striatum (selection pathway) stimulates the striatum to inhibit the substantia nigra pars reticulata (SNr) and the globus pallidus internus (GPi). Here the most relevant signal/action in the current context is filtered, as the SNr and GPi serve as the output of the BG. Because the SNr and GPi inhibit the thalamus, the signal for the selected action should be zero, disinhibiting the neurons associated with that action. To ensure that only one action is completely disinhibited, the STN sends a broad excitatory signal to the SNr and GPi. Furthermore, the activation of D2 receptors results in disinhibition by inhibiting the striatum, which inhibits the globus pallidus pars externus (GPe). The result of an active control pathway is a more active GPe, which inhibits the SNr, GPi, and STN. Therefore, the control pathway stabilizes the system as it modulates the excitatory activity of the STN. Consequently, the dopaminergic modulation in both pathways (D1 and D2) work synergistically in the process of action selection.

Deep brain stimulation (DBS) has proven to be an effective treatment for symptoms of different disorders such as major depression, obsessive-compulsive disorder (OCD), and Parkinson's disease. DBS requires surgery in which electrodes are implanted in a target brain area, typically the basal ganglia in Parkinson's patients. Electrodes are subsequently activated to inject electrical impulses into the target brain area. In this paper, we focus on DBS in both the STN and GPi because they are the most commonly targeted locations for Parkinson's patients, and because there is no clear evidence that DBS is more effective in one vs. the other (Tan et al., 2016). DBS in both the STN and GPi has been shown to significantly improve motor function (Liu et al., 2014), but have differing effects on speech, the relief of depression symptoms, and the need for additional drug treatment (Anderson et al., 2005; Tan et al., 2016). Though we know that Parkinson's patients show abnormally patterned activity in both the GPi and the STN, it is not yet clear whether the electrical impulses injected through DBS excite neurons, inhibit them, block their depolarization, or modulate pathological network activity (McIntyre et al., 2004; Agnesi et al., 2013). It remains an open question whether DBS reduces the overall activity of these neurons or modulates their activity some other way resulting in a more physiological pattern.

In this study, we implement a computer model of the cortico-basal ganglia-thalamus-cortex circuit and use that model to simulate a syllable sequencing task. Unlike other basal ganglia-thalamus models concentrating on speech and syllable sequencing (Civier et al., 2013), our model can be systematically altered to test the effects of external inhibition and low dopaminergic activity. We use the model to investigate the effects of inhibiting the GPi or STN coupled with reduced dopaminergic activity in the SNc. The BG plays an important role in the process of selecting the right action for a current context, so we decided to focus on speech, because the process of speech is a good example of motor planning and action or motor execution. Especially the syllable sequencing task indicates the functionality of the BG in our model.

## The neural model

### The neural engineering framework and semantic pointer architecture

To our knowledge, the Neural Engineering Framework (NEF) (Eliasmith, 2013; Stewart and Eliasmith, 2014) offers the only approach for quantitatively modeling human behavior with large-scale brain models (Eliasmith et al., 2012) composed of spiking neurons. Here, LIF neurons are grouped into ensembles which represent information in the form of vectors of real numbers. These encodings are transformed through connections between ensembles, which are determined automatically through least squares optimization based on the desired function computed between ensembles. One parameter varying between −1 and 1 defines a neural connection as inhibitory or excitatory and describes the strength of connection. The Semantic Pointer Architecture (SPA; see Eliasmith, 2013; Stewart and Eliasmith, 2014) proposes a method of encoding semantic information with vectors, and a set of functions for manipulating those vectors, which allows for large-scale cognitive models in networks of spiking neurons.

In the SPA, semantic information is represented by a vector called a semantic pointer. Semantic pointers represent complex sensory (input), motor (output), or cognitive states like the sensory, motor or linguistic (i.e., phonological) representation of a syllable. Semantic pointers are represented as neural activity patterns occurring in a group of ensembles called a buffer. Buffers can transmit their semantic pointer activity to other buffers through feedforward connections between the ensembles in the buffers. A recurrent connection between a buffer and itself enables short-term memory of a semantic pointer. More complex feedforward connections can bind two semantic pointers together. Finally, the SPA includes basal ganglia and thalamus models, which implement action selection and sequencing.

**Figure 1:**
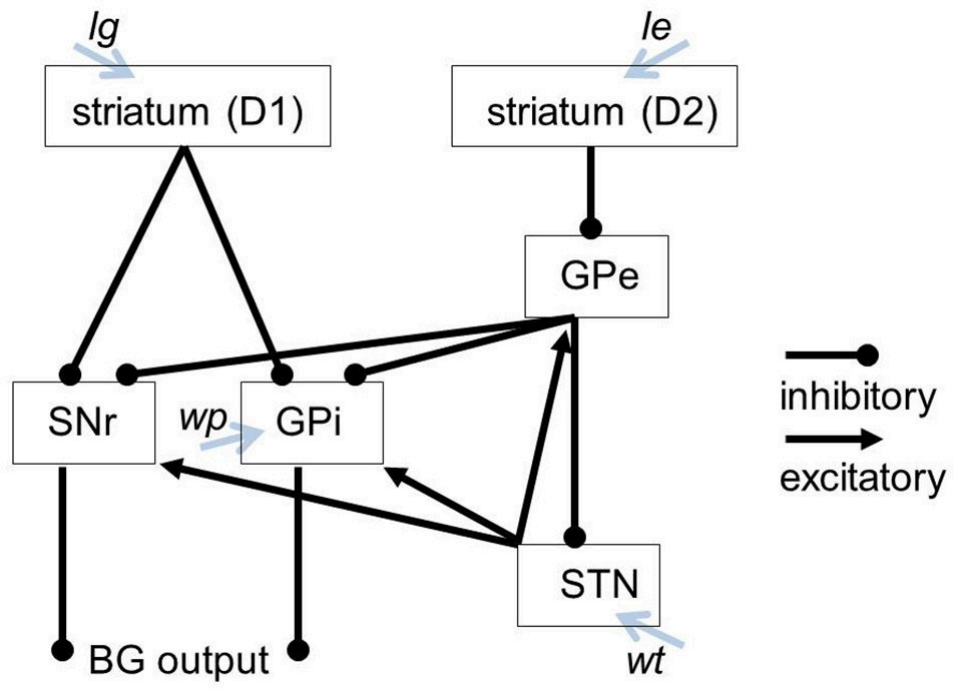
Our BG model including the parameters influencing specific modules of the BG.

The basal ganglia model used in the SPA (Stewart et al., 2010) is based on the approach in Gurney et al. (2001a,b). Important parameters for this model include *lg* (λ_g_ in Gurney et al., 2001b), *le* (λ_e_ in Gurney et al., 2001b), and *wt*. The *lg* and *le* parameters adjust the degree of tonic dopamine within the D1 and D2 modulated pathways, respectively. The *wt* parameter adjusts the strength of afferents to the STN. In the original model, GPi and SNr are combined, as they receive the same inputs and produce the same outputs. To adjust the activity of the SNr and GPi independently, we modified the original model by splitting the GPi and SNr into separate components. This split also required adding a *wp* parameter that influences the activity of the GPi specifically. The modified structure of the BG model and the parameters influencing specific modules of the BG are displayed in Figure 1.

Changing the *lg* and *le* parameters changes the dopamine levels at the striatum and range from typical levels (*le* = *lg* = 0.2) to no dopamine at all (*le* = *lg* = 0). Changing the *wt* parameter scales the synaptic strength of afferents to the STN, where 1 represents the standard weight. Decreasing the *wt*-value leads to a less active STN, resulting in less excitatory inputs to the SNr and GPi, and therefore less inhibition in the thalamus. The *wp* parameter governs the synaptic strength of afferents to the GPi, and ranges from 0 (no activity) to 0.9 (maximum activity). Accordingly modifying the values of the wt or wp parameter conditionally leads to different STN/GPi activity levels. The interconnections of the model as well as the targets of the parameters are illustrated in Figure 1.

### The architecture of the syllable sequencing model

**Figure 2:**
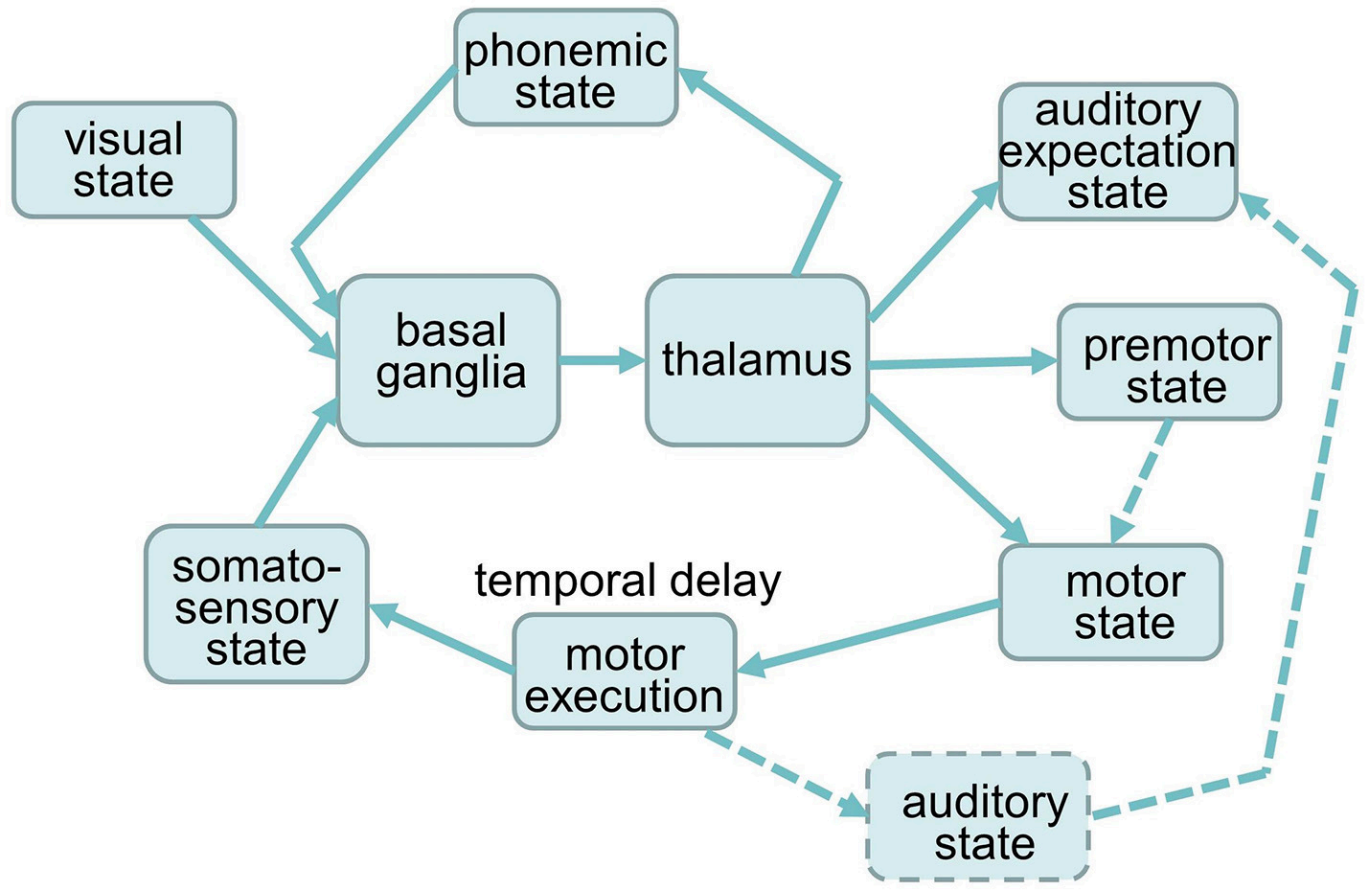
Architecture of the neural model for syllable sequencing. Dashed components are not implemented yet.

To simulate the effects of decreased activity in the SNc, STN, and GPi on syllable sequencing, we used the NEF and the SPA to construct a syllable sequencing model including a cortico-BG-Thal-cortical circuit for action selection and action sequencing. Building on previous work (Senft et al., 2016), we first separated the GPi and SNr, as described above. The overall model architecture is depicted in Figure 2. The model is made up of six cortical buffers representing visual, phonemic, somatosensory, premotor, auditory and motor states, one peripheral motor execution module, and the BG-Thal module.

Predefined semantic pointers represent the states of six syllables. The pointers and their corresponding activity patterns are labeled as BA, DA, GA, PA, TA, and KA and are used to represent the visual, phonological, premotor and auditory expectation state of the labeled syllable. While these pointers are predefined (i.e., learned during speech acquisition), the strength of the neural activity (i.e., the magnitude of the vector) results from the interplay of the basal ganglia with these cortical buffers. When a syllable is executed, the pointer < syllable>_EXEC is activated, carrying information about the motor action and the somatosensory feedback of that syllable. The same pointers are passed to different buffers (e.g., the same BA pointer is used in the phonologicial, premotor, and auditory state buffers). The NEUTRAL pointer represents the case of “no speech activity” and leads to no syllable activating within a cortical buffer. The visual state represents the high-level state of visual input following visual discrimination. The visual state can also have state ZERO, which corresponds to an empty screen. The six syllable pointers represent the other possible visual inputs, i.e., if a syllable is presented on the screen.

Action selection is modeled in the SPA by first defining all possible actions and the situations in which those actions are activated. In the syllable sequencing model, these actions are:

- Set the phonemic buffer to syllable BA, if the visual buffer is activated with BA
- Set the phonemic buffer to NEUTRAL if the visual buffer is activated with ZERO
- Set the premotor buffer and auditory expectation buffer to the syllable to BA, DA etc. and set the motor buffer to the corresponding syllable execution pointer (e.g., BA_EXEC, DA_EXEC), if the phonemic buffer is activated to BA, DA etc.
- Set the phonemic buffer to the next syllable (e.g., DA) if the somatosensory buffer contains the preceding syllable (e.g., BA_EXEC). The order of syllables is BA, DA, GA, PA, TA, KA.

These actions (following the “set …”) resulting from conditions (following the “if …”) are represented by the cortical buffers (Figure 2). Thus, “action channels” are modeled, one for each excitatory or inhibitory pathway defined in Figure 1 between the parts or components of the basal ganglia. It should be noted that there are far more action channels between cortex and striatum and subsequently between all parts of the BG, but our model is as small as can implement this task in which the subject would be given the command “start speaking if you see the syllable BA on the screen and perform a syllable sequencing in the order BA, DA, GA, PA, TA, KA, and so on.” The strength of the signal within each of these channels is called “utility” (Stewart et al., 2010) and the numeric “utility value” or activation level within each of the action channels is modified by the basal ganglia such that the action with the highest utility value is disinhibited. With the action activation conditions set, the different utility values get passed from the cortex (e.g., visual state, somatosensory state and phonemic state) to the BG. The channel forwarding the strongest (most relevant in the current context) neural signal from the striatum to the SNr and GPi leads to the highest disinhibition (inhibiting the inhibitory influence of SNr and GPi on the thalamus). Therefore, this action is selected and passed on, when its inhibitory signal from SNr and GPi on the thalamus is zero. This can be seen as a winner-take-all mechanism. When there is strong inhibition from the striatum, there is a corresponding amount of broad excitation from the STN. Both inputs are summed in the SNr and GPi so that only one channel exerts inhibitory influence on the thalamus. This is how the BG determines the appropriate single action (action selection) before passing it on to the thalamus. In order to adjust to circumstances where there are many actions with large or low utilities, Gurney et al. (2001a,b) thought of the GPe as being well suited to modulating the rest of the BG such that close utility values can be differentiated. The GPe (like the SNr and GPi) receives excitatory input from the STN and inhibitory input from the D2 cells in the striatum. The resulting GPe activity inhibits the GPi, STN and SNr. This inhibition functions as a control signal to attenuate the excitation in the STN such that only a single action is selected. Through this regulatory influence the action selection functions across a wide range of utility values. All together the control pathway may increase the contrast between the utility values and stabilizes the system.

It should be noted that in a full model of syllable sequencing and production, the premotor state buffer would lead to a sequence of low-level motor states articulating the syllable. In this model, we only generate the go-signal for motor execution instead of the detailed motor information required for syllable production. Moreover, in a full model, the articulation would result in a feedback auditory state. This feedback auditory state would be compared with the pre-activated auditory expectation state for that syllable. However, auditory signal generation is beyond the scope of the current paper as it is not required to simulate the pathological behavior seen in Parkinson's patients in syllable sequencing tasks.

## Methods: simulations and evaluation

Seven hundred and fifteen simulation trials were run using the syllable sequencing model described above implemented in Nengo (Bekolay et al., 2014). Four parameters are used to test different aspects of the model during these trials: varying the *lg* and *le* parameters simulate different dopamine levels (*lg* and *le* are always set to the same value); varying the *wt* parameter simulates different STN activity levels; and varying the *wp* parameter simulates different GPi activity levels.

The syllable sequencing task (described in detail in section The Architecture of the Syllable Sequencing Model) involves repeating six syllables, /ba/, /da/, /ga/, /pa/, /ta/ and /ka/. The model repeats this sequence of syllables as often as possible until the simulation time (5.5 s) ends. Syllable production starts when the visual state represents the pointer BA. The motor execution module (see Figure 2) starts the production of syllable /ba/ by S-pointer pulse BA_EXEC. Following activation of the motor execution model, we explicit delay activity for 200 ms to emulate articulation of the syllable, as somatosensory feedback would not be available until the syllable is articulated. The resulting activity in the somatosensory state buffer can be thought of as a kind of feedback that signals the end of the current syllable. After activation of this feedback signal, the process of initiating the next syllable begins, which takes about 80 ms from the activation of the cortical state to the disinhibition of the correct action in the thalamus. Thus, with the explicit 200 ms delay and loop time of 80 ms, the total duration that the model spends in each syllable in around 280 ms.

Two sets of simulations were carried out, one to measure the effect of decreased activity in the STN and one to measure the effect of decreased activity in the GPi. For the first set, the activity of the STN was measured for 8 levels: *wt* = 1.0, 0.95, 0.9, 0.8, 0.6, 0.4, 0.2, 0. For the second set, the activity of the GPi was measured for 7 levels: *wp* = 0.9, 0.75, 0.6, 0.45, 0.3, 0.15, 0. The maximum activity level of GPi occurs when *wp* = 0.9 (see Gurney et al., 2001b, p. 417), while the maximum activity level for STN occurs when *wt* = 1.0. We ran of trials with *wt-*values of 0.95 and 0.9 because we hypothesized that errors in syllable sequencing would occur at high levels of *wt* activity. For both sets, we ran trials with 11 levels of dopamine: *le* = *lg* = 0.20, 0.18, 0.16, …, 0.04, 0.02, 0. In total, 88 parameter values were simulated in the first set of experiments, and 77 parameter values were simulated in the second set. Each experiment was simulated five times with different randomly generated neural parameters (randomly generated semantic pointers and covarying synaptic link weights, leading to five different exemplars of the model), resulting in 440 trials for the first set of experiments, and 385 trials for the second set. Each trial was simulated for 5.5 simulated second. There exists variability between trials because on each trial Nengo generates a new set of randomly sampled neuron parameters and solves for new sets of connection weights to implement the model as best as possible given the random neuron parameters.

Quantitative and qualitative analyses of simulation results were done by interpreting the similarity between the activity of the motor buffer and the activity evoked by each pointer (see Figure 2). The given similarity values reflect current activity in a specific neural buffer. This motor buffer was chosen for analysis because the activity of this buffer leads directly to syllable execution. Quantitative analysis was done by counting the number of correctly sequenced syllable pulses. The number of correctly sequenced syllables was summed across the five trials for each set of parameter values. Because each trail begins with 0.5 s with no input, the total task execution time was 5 × 5 = 25 s for each set of parameter values.

In addition it should be stated that in our network model, each buffer consists of 32 one-dimensional ensembles with 50 neurons in each ensemble (1600 neurons total). Ensembles in the basal ganglia and thalamus also consist of 50 neurons each, yielding 2100 neurons in the basal ganglia and 400 neurons in the thalamus. The BG model is described in detail in Eliasmith et al. (2012). Our model consists of 13,300 spiking neurons total (2,500 for BG and Thal, 1,600 for each of 7 buffers).

## Results

**Figure 3:**
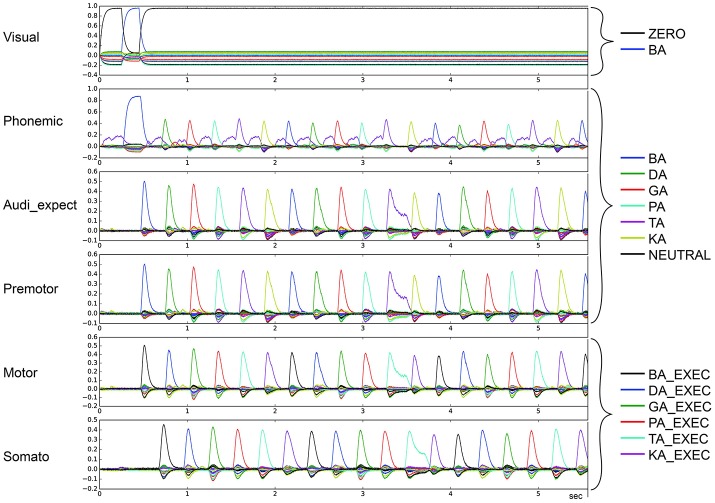
Similarity between neural activity patterns of buffers and neural activity patterns of predefined semantic pointers representing syllable activations at visual buffer, phonemic buffer, auditory expectation buffer, premotor buffer, motor buffer and somatosensory buffer. Normal case: Normal syllable sequencing (*le* = *lg* = 0.2; *wt* = 1.0, *wp* = 0.9). Different colors indicate similarity with different syllables. Syllable execution delay time is 200 ms. (Cortical syllable pointers and syllable execution pointers show different colors for one syllable).

Figure 3 depicts the output of the model in non-pathological cases. The vertical axis represents the similarity (dot product) between the output of a buffer and a given semantic pointer. All possible pointers are plotted.

The input to the model is provided to the visual input buffer. A visual input of BA is provided for the first 200 ms and is ZERO for all other time periods. The visual state BA (i) activates the phonemic state BA via the cortico-cortical BG-thalamus loop, which (ii) sets the premotor and auditory expectation buffers to BA, and sets the motor state buffer to BA_EXEC. This represents the first two action selection processes performed by the cortico-BG-Thal-cortex loop. Action selection is simple in these two cases because only the semantic pointer representing the syllable BA is active in the visual buffer or in the phonemic buffer respectively. This leads to a strong and lasting phonemic activation pattern for the syllable BA for sequence initiation in comparison to other phonemic activation patterns.

Following these two actions, the motor execution module feeds the BA_EXEC pointer back to the somatosensory buffer with a delay of 200 ms. This triggers the next action, (iii) the activation of the next syllable, DA, in the phonemic buffer. The sequence then proceeds as with BA, only with the syllable DA, leading to the activation of GA, and so on.

For illustrative purposes, let us consider the point in time when the activation of the visual input BA starts. At this point, BA is not active in the phonemic buffer and BA_EXEC is not active in the somatosensory buffer. Thus, only the action “activate syllable BA in phonemic buffer” has a high utility value and will be selected. Once the visual input ends, the most active buffer is the phonemic buffer and still no activity occurs in the somatosensory buffer. Thus, the next action selected is “activate the syllable BA in the auditory expectation, premotor and motor buffers.” Once the activity of BA_EXEC is sufficiently strong in the somatosensory buffer, the action “select next syllable (DA) within phonemic buffer” is selected.

**Figure 4:**
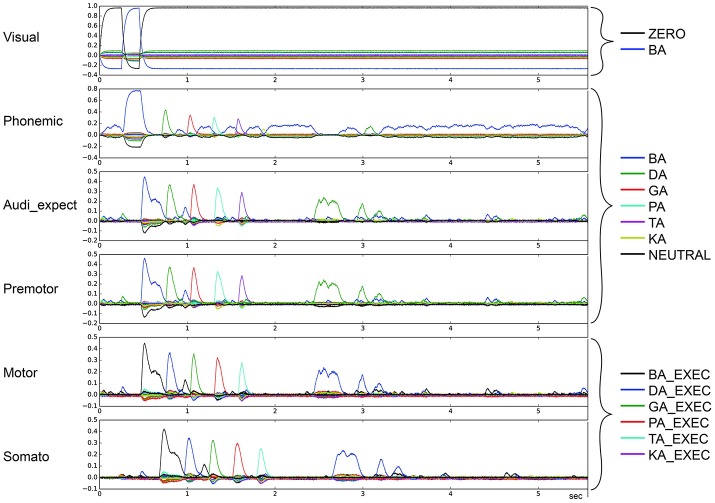
Similarity between neural activity patterns of buffers and neural activity patterns of predefined semantic pointers representing syllable activations at different cortical buffers (cf. Figure 3). In this case, there is a break of syllable sequencing followed by repetitions (*le* = *lg* = 0.14; *wt* = 0.9, *wp* = 0.9). Syllable execution delay time is 200 ms.

**Figure 5:**
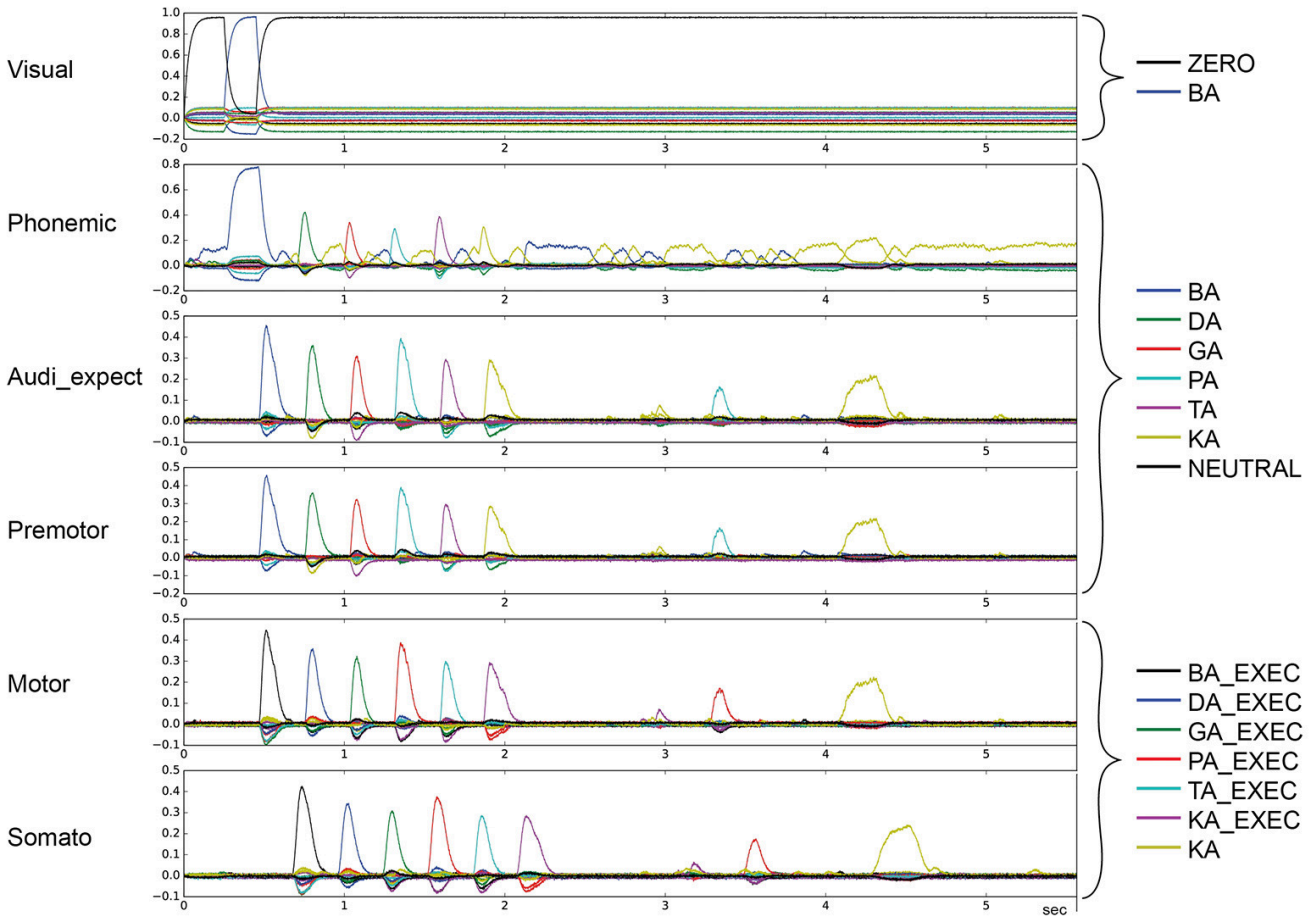
Similarity between neural activity patterns of buffers and neural activity patterns of predefined semantic pointers representing syllable activations at different cortical buffers (cf. Figure 3). In this case, there is a break of syllable sequencing followed by non-ordered syllables (skipping of syllables) (*le* = *lg* = 0.16; *wt* = 1.0, *wp* = 0.9). Syllable execution delay time is 200 ms.

**Figure 6:**
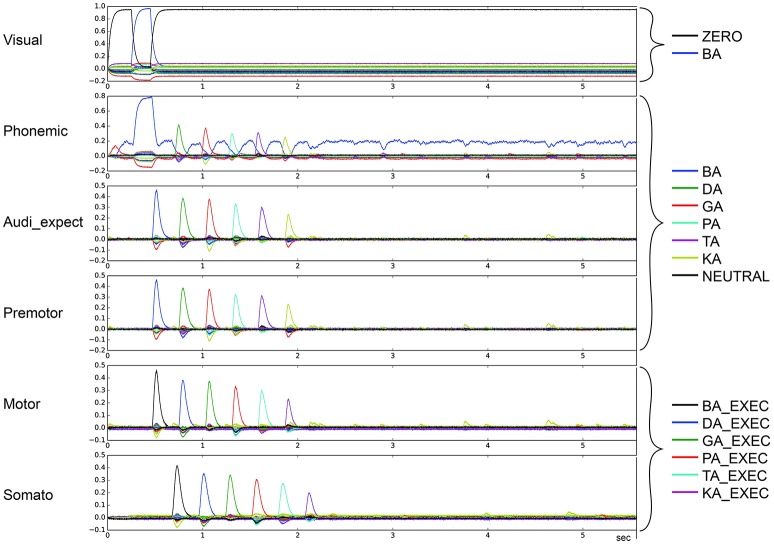
Similarity between neural activity patterns of buffers and neural activity patterns of predefined semantic pointers representing syllable activations at different cortical buffers (cf. Figure 3). In this case, there is a break of syllable sequencing followed by no further syllables (*le* = *lg* = 0.14; *wt* = 0.9, *wp* = 0.9). Syllable execution delay time is 200 ms.

To categorize the overall effects of STN and GPi activity levels on syllable sequencing, we also performed two qualitative analyses. In the first qualitative analysis, we classified error trials as belonging to one or more of four categories of errors. *Repetition* errors occur when the same syllable is repeated multiple times; see Figure 4 in which /da/ is repeated. *Skipping* errors occur when syllables activate in the wrong order, as in Figure 5. *Restart* errors occur when the syllable sequence stops then restarts after a short pause of around 100 ms, as can be seen in Figures 4, 5. Finally, *halting* errors occur when, the sequence stops and no further syllables are produced, as in Figure 6. We consider syllable sequencing without such errors to be firmly established (stable).

These four types of errors result from the same underlying neural processes, specifically the effects of reduced dopamine on the BG's action selection process. In the case of repetition and skipping errors, the somatosensory state buffer signals that the next syllable can be activated, but the BG selects the wrong action. In the case of halting and restarting errors, the signal coming from the somatosensory state buffer results in no action being selected in the BG. Errors in the BG's action selection process typically result from utility values that are too close. It is therefore likely that the modified striatal activity that results from reduced dopamine levels either directly modifies input utility values, or interferes with the control pathways that help differentiate between close utility values.

**Figure 7:**
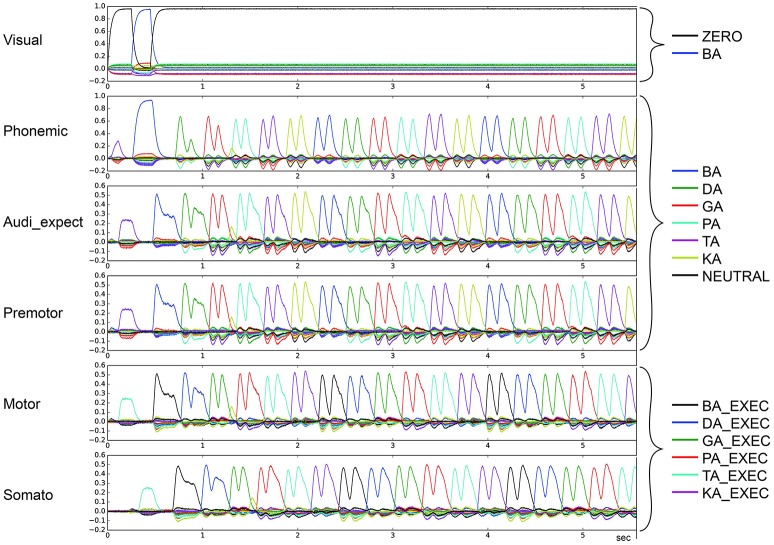
Similarity between neural activity patterns of buffers and neural activity patterns of predefined semantic pointers representing syllable activations at different cortical buffers (cf. Figure 3). In this case, we see oscillatory behavior in which there are two activations of each syllable (*le* = *lg* = 0.16; *wt* = 0.2, *wp* = 0.9). Syllable execution delay time is 200 ms.

**Figure 8:**
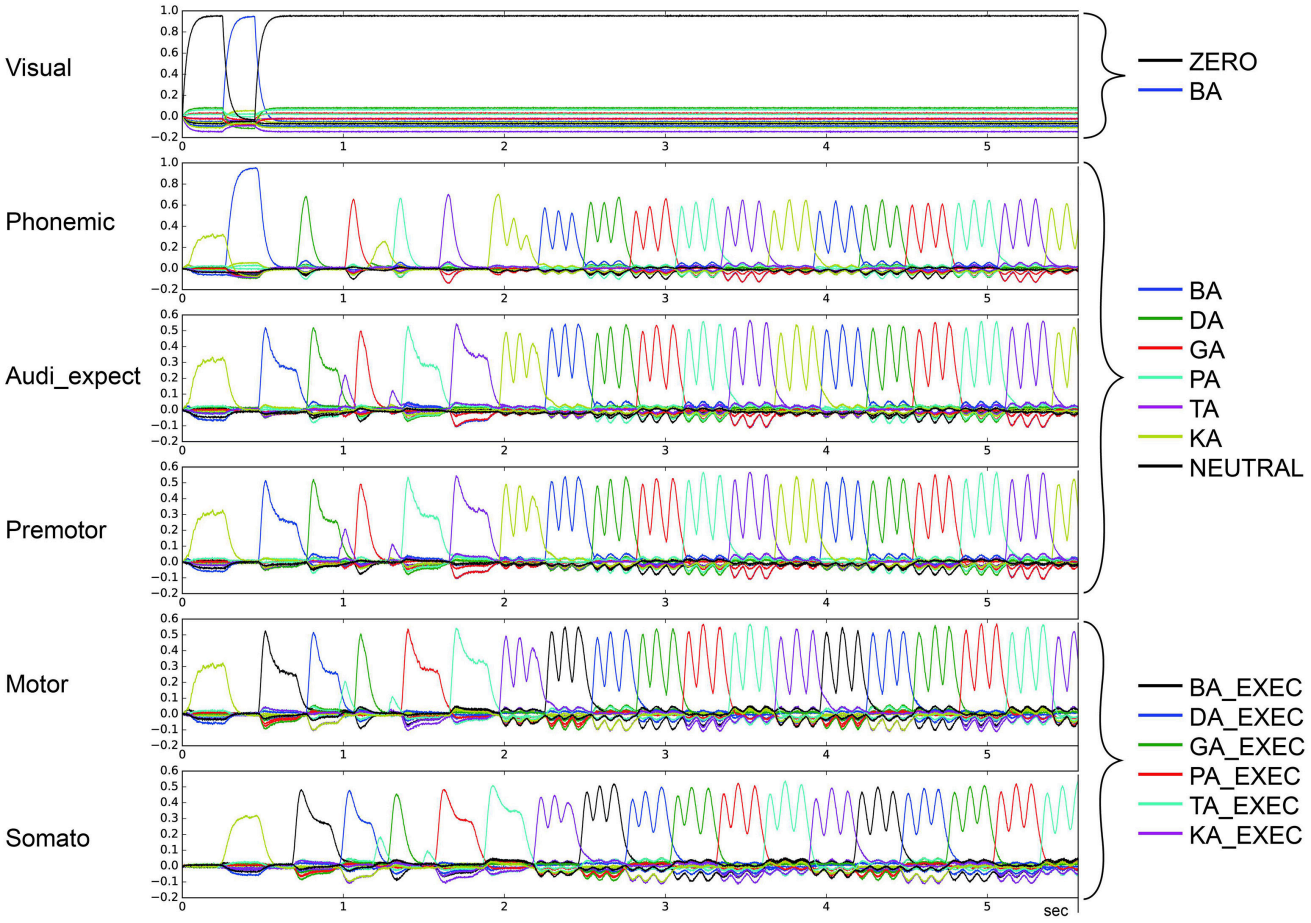
Similarity between neural activity patterns of buffers and neural activity patterns of predefined semantic pointers representing syllable activations at different cortical buffers (cf. Figure 3). In this case, we see oscillatory behavior in which there are three activations of each syllable (*le* = *lg* = 0.16; *wt* = 0.2, *wp* = 0.9). Syllable execution delay time is 200 ms.

**Figure 9:**
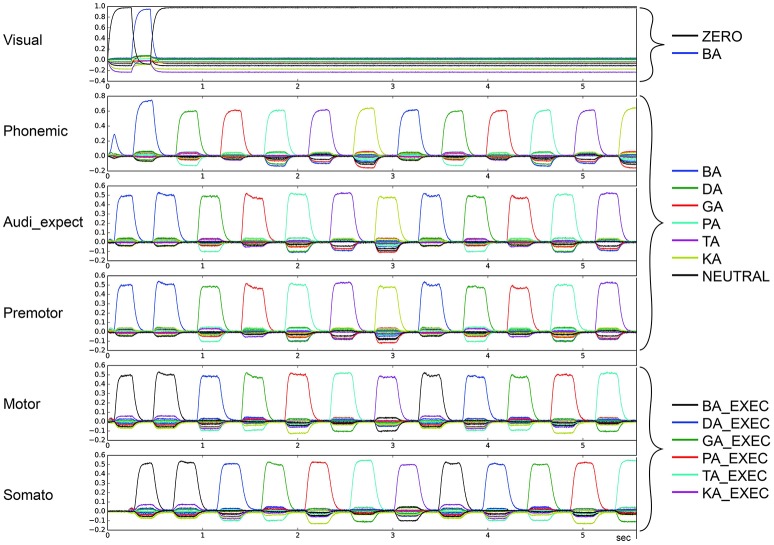
Similarity between neural activity patterns of buffers and neural activity patterns of predefined semantic pointers representing syllable activations at different cortical buffers (cf. Figure 3). In this case, there is a broad peak for each syllable (*le* = *lg* = 0.08; *wt* = 1.0, *wp* = 0.15). Syllable execution delay time is 200 ms.

In the second qualitative analysis, we inspected the shape of the syllable pointer similarity levels at the motor buffer. Typically, these pulses exhibit a tall slim single peak with duration no longer than 100 ms followed by a no-peak interval of about 300 ms. However, in some cases a “degenerate” syllable pulse occurs, either exhibiting multiple peaks (*oscillatory* behavior; see Figures 7, 8) or one broad peak with a duration up to 300 ms followed by a no peak interval of about 100 ms (*broad* peak behavior; see Figure 9). Both can result in a non-monotonic behavior regarding the number of correctly sequenced syllables.

Tables 1A, 2A list the total number (sum) of correctly sequenced syllables over all 5 trials. Tables 1B, 2B list the mean and variance of the number of correctly sequenced syllables per trial. Tables 1A,B list the results for the first set of experiments, which vary STN activity and dopamine level. Tables 2A,B list the results for the second set of experiments, which vary GPi activity and dopamine level.

**Table 1A:** Sum of number of correctly sequenced syllables in five trials for a given parameter combination *lg* = *le* vs. wt (experiment 1).

**le/lg wt**	**0.20**	**0.18**	**0.16**	**0.14**	**0.12**	**0.10**	**0.08**	**0.06**	**0.04**	**0.02**	**0.00**
1.0	**90**	**80**	**27**	**17**	**13**	**9**	**9**	**6**	**6**	**5**	**5**
0.8	**90**	**90**	**90**	**71**	**56**	**17**	**11**	**8**	**8**	**5**	**5**
0.6	**86**	**75**	**90**	**90**	**90**	**75**	**36**	**13**	**9**	**9**	**5**
0.4	**86**	**86**	**59**	**90**	**90**	**90**	**71**	**34**	**14**	**9**	**8**
0.2	**85**	**85**	**85**	**70**	**89**	**58**	**90**	**80**	**45**	**22**	**11**
0.0	**85**	**69**	**85**	**70**	**85**	**55**	**90**	**41**	**42**	**33**	**17**

*Colors indicate the region of values (compare with Table 3). Rows: decreasing activity of STN (wt decreasing); columns: decreasing dopamine level (lg = le)*.

**Table 1B:** Mean plus or minus variance of the number of correctly sequenced syllables for each trial for a given combination of *lg* = *le* and *wt-*values.

**le/lg wt**	**0.20**	**0.18**	**0.16**	**0.14**	**0.12**	**0.10**	**0.08**	**0.06**	**0.04**	**0.02**	**0.00**
1.0	18.0 (±0.0)	16.0 (±4.5)	5.4 (±2.3)	3.4 (±1.1)	2.6 (±0.5)	1.8 (±0.4)	1.8 (±0.4)	1.2 (±0.4)	1.2 (±0.4)	1.0 (±0.0)	1.0 (±0.0)
0.8	18.0 (±0.0)	18.0 (±0.0)	18.0 (±0.0)	14.2 (±5.3)	11.2 (±5.5)	3.4 (±0.9)	2.2 (±0.4)	1.6 (±0.5)	1.6 (±0.5)	1.0 (±0.0)	1.0 (±0.0)
0.6	17.2 (±0.4)	15.0 (±6.7)	18.0 (±0.0)	18.0 (±0.0)	18.0 (±0.0)	15.0 (±5.7)	7.2 (±6.1)	2.6 (±0.5)	1.8 (±0.4)	1.8 (±0.8)	1.0 (±0.0)
0.4	17.2 (±0.4)	17.2 (±0.4)	11.8 (±8.5)	18.0 (±0.0)	18.0 (±0.0)	18.0 (±0.0)	14.2 (±5.5)	6.8 (±5.3)	2.8 (±1.8)	1.8 (±0.4)	1.6 (±0.9)
0.2	17.0 (±0.0)	17.0 (±0.0)	17.0 (±0.0)	14.0 (±6.7)	17.8 (±0.4)	11.6 (±8.8)	18.0 (±0.0)	16.0 (±4.5)	9.0 (±7.1)	4.4 (±0.5)	2.2 (±0.4)
0.0	17.0 (±0.0)	13.8 (±7.2)	17.0 (±0.0)	14.0 (±6.7)	17.0 (±0.0)	11.0 (±8.2)	18.0 (±0.0)	8.2 (±8.5)	8.4 (±7.2)	6.6 (±1.8)	3.4 (±1.7)

*Colors here indicate oscillatory behavior of syllable pulses at the level of motor buffer (light green) and irregularity in syllable sequencing following a break in normal syllable sequencing (yellow). It should be noted that high mean values with 0 variance occur when there are no syllable sequencing errors*.

**Table 2A:** Number of correctly sequenced syllables in five trials for a given parameter combination *lg* = *le* vs. wp (experiment 2).

**le/lg wp**	**0.20**	**0.18**	**0.16**	**0.14**	**0.12**	**0.10**	**0.08**	**0.06**	**0.04**	**0.02**	**0.00**
**0.90**	**90**	**90**	**56**	**14**	**12**	**11**	**9**	**6**	**5**	**5**	**5**
**0.75**	**90**	**90**	**74**	**90**	**36**	**13**	**8**	**7**	**7**	**5**	**5**
**0.60**	**87**	**89**	**89**	**89**	**73**	**53**	**27**	**6**	**7**	**7**	**6**
**0.45**	**88**	**89**	**89**	**88**	**74**	**87**	**70**	**42**	**14**	**16**	**7**
**0.30**	**86**	**86**	**83**	**82**	**81**	**66**	**62**	**63**	**61**	**50**	**29**
**0.15**	**80**	**80**	**80**	**75**	**58**	**69**	**56**	**55**	**55**	**59**	**39**
**0.00**	**76**	**68**	**78**	**58**	**54**	**55**	**55**	**55**	**55**	**52**	**50**

*Colors indicate the region of values (compare with Table 3). Rows: decreasing activity of GPi (wp decreasing); columns: decreasing dopamine level (lg = le)*.

**Table 2B:** Mean plus or minus variance of the number of correctly sequenced syllables for each trial for a given combination of *lg* = *le* and *wp*-values.

**le/lg wp**	**0.20**	**0.18**	**0.16**	**0.14**	**0.12**	**0.10**	**0.08**	**0.06**	**0.04**	**0.02**	**0.00**
0.90	18.0 (±0.0)	18.0 (±0.0)	11.2 (±5.4)	2.8 (±1.3)	2.4 (±0.9)	2.2 (±0.4)	1.8 (±0.8)	1.2 (±0.4)	1.0 (±0.0)	1.0 (±0.0)	1.0 (±0.0)
0.75	18.0 (±0.0)	18.0 (±0.0)	14.8 (±7.2)	18.0 (±0.0)	7.2 (±6.1)	2.6 (±2.1)	1.6 (±0.5)	1.4 (±0.5)	1.4 (±0.5)	1.0 (±0.0)	1.0 (±0.0)
0.60	17.4 (±0.5)	17.8 (±0.4)	17.8 (±0.4)	17.8 (±0.4)	14.6 (±7.1)	10.6 (±8.5)	5.4 (±6.7)	1.2 (±0.4)	1.4 (±0.5)	1.4 (±0.5)	1.2 (±0.4)
0.45	17.6 (±0.5)	17.8 (±0.4)	17.8 (±0.4)	17.6 (±0.5)	14.8 (±6.6)	17.4 (±0.9)	14.0 (±6.7)	8.4 (±7.5)	2.8 (±0.8)	3.2 (±3.3)	1.4 (±0.5)
0.30	17.2 (±0.4)	17.2 (±0.4)	16.6 (±0.5)	16.4 (±0.9)	16.2 (±0.8)	13.2 (±6.3)	12.4 (±1.9)	12.6 (±1.1)	12.2 (±4.0)	10.0 (±4.2)	5.8 (±4.9)
0.15	16.0 (±0.7)	16.0 (±0.7)	16.0 (±0.7)	15.0 (±1.2)	11.6 (±1.3)	13.8 (±1.9)	11.2 (±0.4)	11.0 (±0.0)	11.0 (±0.0)	11.8 (±1.8)	7.8 (±4.4)
0.00	15.2 (±2.5)	13.6 (±2.4)	15.6 (±1.8)	11.6 (±1.3)	10.8 (±0.4)	11.0 (±0.0)	11.0 (±0.0)	11.0 (±0.0)	11.0 (±0.0)	10.4 (±0.5)	10.0 (±0.0)

*Colors here indicate oscillatory behavior of syllable pulses at the level of motor buffer (light green), slow but stable syllable sequencing (dark green) and irregularity in syllable sequencing following a break in normal syllable sequencing (yellow). It should be noted that high mean values with 0 variance occur when there are no syllable sequencing errors*.

Table 3 provides a legend for the colors used in Tables 1A, 2A. Each color indicates a different range of correctly sequenced syllables. In both sets of experiments, we can see that decreased STN or GPi activity results in correct syllable sequencing despite decreased dopamine levels. For normal levels of STN or GPi activity (*wt* = 1.0, *wp* = 0.9), reduced dopamine levels lead to decreased correct syllable sequencing starting with reduction of about 20% (*le* = *lg* = 0.16). With reduced GPi or STN activity, syllable sequencing is not impacted until very low dopamine levels. A statistical significant decrease in correctly sequenced syllables occurs between *le* = *lg* = 0.20 and *le* = *lg* = 0.16 for *wt* = 1.0 (t = 11.06, *p* < 0.001, one-sided *t*-test) as well as for *wp* = 0.9 (t = 2.81, *p* < 0.05).

**Table 3:** Specification of 6 different regions for different levels of numbers of correctly sequenced syllables as marked by different colors in Tables 1A, 2A concerning number of region, color of region and maximum and minimum number of correctly sequenced syllables in a region.

**Number of region**	**Color**	**Maximum number of correctly sequenced syllables**	**Minimum number of correctly sequenced syllables**
1	dark red	90	75
2	red	74	61
3	light red	60	45
4	light blue	44	31
5	blue	30	15
6	dark blue	14	0

Looking at the first set of experiments (Table 1), we can see that reducing STN activity by about 20% (*wt* = 0.8) results in a strong decrease in correct syllable sequencing not until an over 50% reduction of dopamine (*le* = *lg* = 0.10). A statistical significant decrease in correctly sequenced syllables occurs between *le* = *lg* = *0.20* and *le* = *lg* = 0.10 for *wt* = 0.8 (t = 28.24, *p* < 0.0001, one-sided *t*-test), while the change in correctly sequenced syllables between *le* = *lg* = *0.20* and *le* = *lg* = *0.16* is not significant for *wt* = 0.8 (*t* = 1.0, *p* > 0.05, one-sided *t*-test). Thus, the model predicts that a protocol that results in a 20% reduction in STN activity can compensate for a significant reduction in tonic dopamine levels.

We can also see that for even lower STN activity levels, correct syllable sequencing still occurs at very low dopamine levels (*le* = *lg* = 0.06; see Table 1). Here, the difference in the number of correctly sequenced syllables for *le* = *lg* = 0.20 is not significantly different from *le* = *lg* = 0.06 for *wt* = 0.2 (*t* = 0.57, *p* > 0.05, one-sided *t*-test one-sided).

Looking at the second set of experiments (Table 2), if GPi activity is reduced by about 33% (*wp* = 0.6) we see a strong decrease in correct syllable sequencing not until a dopamine reduction of about 60% (*le* = *lg* = 0.08), compared to 20% for maximum GPi activity. A significant decrease in correctly sequenced syllables occurs between *le* = *lg* = 0.20 and *le* = *lg* = 0.08 for *wp* = 0.6 (*t* = 4.35, *p* < 0.01, one-sided *t*-test) while no significant decrease occurs for *le* = *lg* = 0.20 and *le* = *lg* = 0.16 for *wp* = 0.6 (*t* = 1.27, *p* > 0.05, one-sided *t*-test). Thus, the model predicts that a protocol that results in a 33% reduction in GPi activity can compensate for a significant reduction in tonic dopamine levels.

A closer look at Tables 1, 2 indicates that stable syllable sequencing (stable syllable sequencing for more than 83% of total task execution time) occurs in 26 of 66 (39%) parameter sets modifying STN activity and in 25 of 77 (32%) parameter sets modifying GPi activity. Mildly distorted syllable sequencing (stable syllable sequencing for more than 50% of the total task execution time) occurs in 36 of 66 (55%) parameter sets modifying STN activity and for 51 of 77 (66%) parameter sets modifying GPi activity.

The maximum number of correctly sequenced syllables in 25 s is 90 (278 ms per syllable), which occurs for dopamine level *lg* = *le* = 0.2 and fully active STN and GPi (see Figure 4). Because the completion of a correct syllable sequence is signaled by the somatosensory buffer 200 ms after the occurrence of the syllable in the motor buffer, a mean interval of about 80 ms is needed to initiate the next syllable; i.e., each loop of our cortico-BG-Thal-cortical network takes around 80 ms to complete end-to-end. The 80 ms loop time can be broken down into smaller parts of the loop. It takes between 15 and 40 ms for the BG to select an action. The speed of action selection depends on the difference between the highest utility value and other utility values; that is, when only a single action has high utility, it can be selected quickly, but when multiple actions have similarly high utility, it takes time for the BG to determine which action has highest utility (see Stewart et al., 2010). Communication of a pointer from one state buffer to another consistently takes around 15 ms. Since the longest sequence of buffers is four, this contributes 60 ms to the loop time. As a result, a loop can take as short as 75 ms or as long as 100 ms. Since the mean interval is 80 ms, we can conclude that most actions are selected quickly, with one action having clearly higher utility than the other actions.

The results of our qualitative analysis are given in Table 1B for the first set of experiments and in Table 2B for the second set. We see that for both experiments, when few syllables are correctly sequenced (correct sequencing is below 50% of total task execution time), it is typically not the result of halting errors (i.e., no further syllable production) but instead irregular syllable sequencing behavior (repetition, skipping, or restarting errors).

For stable syllable sequences (above 50% of total task execution time), aside from repetition errors (Figure 3) we also find oscillatory behavior, with two or three similarity peaks (see Figures 7, 8). This behavior leads to a slight reduction in syllable speed, from sequencing 90 to around 85 syllables in 25 s, a reduction of about 6%. This oscillatory behavior occurs with reduced STN activity (see Table 1B) and reduced GPi activity (see Table 2B). When GPi activity is reduced, we also find broad syllable peaks (see Figure 9), leading to slow syllable sequencing, which we label “*low frequency syllable repetition*” (dark green zones in Table 2B). Here, the syllable speed is reduced from sequencing 90 syllables to about 55, a reduction of about 40%.

## Discussion and conclusions

In this study we modified a model of the BG from Gurney et al. (2001a,b) by splitting the GPi and SNr into two separate modules. We then developed a syllable sequencing model including cognitive, sensory, and motor buffers that are modified by the modified BG model to examine the action selection process for varying levels of dopamine and STN and GPi inhibition. Our simulated decrease in dopamine levels (varying parameters *le* and *lg*) emulates the loss of SNc function seen in Parkinson's patients (Goetz and Pal, 2014). Our simulated decrease in STN and GPi activity levels (varying parameters *wt* and *wp*) emulates one theory of the effect of DBS on the BG.

Our simulations show that, like in some Parkinson's patients, decreasing dopamine levels results in errors in syllable sequencing (at *le* = *lg* = 0.16). Dopamine is crucial for the action selection process because its reduction leads to less inhibition from the striatum and GPe on the SNr and GPi, so that at some point not one of the inhibitory signals of the SNr and the GPi is itself inhibited to zero and therefore no signal is passed on to the thalamus. Irregularities like additional repeated syllables or syllables out of order can occur when the utility values of different actions are too similar. Low dopamine levels may also contribute to the inability to differentiate between close utility values due to weak inhibitory signals on the SNr and GPi.

Our results indicate that decreasing STN or GPi activity restabilizes syllable sequencing even if dopamine levels are dramatically reduced. The action selection process depends on a precise balance between inhibitory and excitatory signals in the BG. Inhibiting the STN or GPi directly compensates for the reduced inhibitory signals on the STN (due to a less active GPe) and GPi (due to a less active striatum) that result from reduced dopamine levels. The inhibited STN has less excitatory influence on the SNr and GPi, so that the inhibitory input they receive becomes sufficient to disinhibit the most relevant action again. With an ongoing reduction of dopamine levels, this inhibitory signal becomes insufficient again and we observe a break in normal syllable sequencing (see Table 1A). Similar applies for GPi inhibition, with an ongoing depletion of dopamine levels the inhibitory signal becomes insufficient (see Table 2A).

However, in our simulations, restabilizing the BG this way alters the activity of the state buffers significantly. With normal dopamine levels, action pulses show a duration of about 100 ms. With reduced dopamine levels combined with STN or GPi activity reduction, these pulses may broaden or oscillate (i.e., pulses show two or three peaks). While lowering activity in the STN or GPi compensates for the decreased inhibition resulting from lowered dopamine levels, it also affects the action selection dynamics.

The GPi and SNr provide BG output by integrating activity from striatum, STN, and GPe. Lowering dopamine levels in this approach affects the input coming from striatum, but not STN and GPe. Therefore, the amount of additional GPi inhibition required to cancel out the effects of reduced dopamine levels depends on utility values, since activity in all parts of the BG depend on utility values. When the external inhibitory influence exceeds a certain limit, GPi can not select the most relevant action, as there are multiple actions being disinhibited (not only the one with the highest utility value). This could be called over-inhibition. It is likely that action selection dynamics are more stable when the GPi is over-inhibited, because an action only gets selected, when it is the only one disinhibited to zero (winner-take-all mechanism). Therefore, the over-inhibited GPi would not select an incorrect action and no error would occur. This over-inhibition, however, means that it takes longer for the recurrent connections between the GPe and STN to accumulate enough activity to overcome the external GPi inhibition and the addition SNr activity resulting from reduced dopamine levels. As can be seen in Table 2, acceptable performance is maintained even at very high levels of GPi inhibition, as the overall BG dynamics are largely unchanged except for the amount of GPi activity necessary to disinhibit an action. That is, inhibiting the GPi generally improves the stability of the BG at the cost of speed.

The STN, on the other hand, is recurrently connected to the GPe. Because of this recurrent connection, inhibiting STN affects both the speed and stability of action selection. When moderately inhibited, it can overcome moderate decreases in dopamine levels while only minimally affecting the speed of action selection because the GPe compensates for decreased STN activity. However, as can be seen in Table 1, when STN activity is inhibited too strongly, the internal BG dynamics break down and cannot recover.

It is interesting to note that as activity and dopamine levels drop, performance moves predictably from typical performance to oscillatory behavior, as happens when *wp* = 0.45 (see Table 2A) and at *wt* = 0.4 (see Table 1A), to slowed behavior. This suggests that oscillatory behavior, which may result in syllable repetitions, occurs when the amount of GPi or STN inhibition is just enough to overcome the reduction in dopamine levels. Therefore, it may be possible to differentiate between STN and GPi inhibition in DBS protocols by increasing the amount of inhibition and observing whether speech sequences halt (as in STN inhibition) or slow down (as in GPi inhibition).

Thus we hypothesize that inhibiting the GPi (and thereby reducing the influencing amount of excitation by the STN on the BG output) may lead to a restabilization process in action selection. However reducing the activity of the GPi (as potentially evoked by DBS) is an imprecise process, and would most certainly effect the action selection calculation. But our idea is that inhibiting the GPi makes up for the lack of inhibition coming from the striatum, but disturbs the action selection calculation. Eventually we would estimate the reduction of activity in the GPi (by DBS) to have a less potent influence on the whole action selection system than the reduction of activity of the STN (by DBS), because in reduction of GPi activity the action selection process would simultaneously be executed in the SNr, which could compensate for disturbances resulting from this activity reduction. Therefore, it can be presumed (and is supported by the simulations reported in this publication) that even in the case of reduced GPi activity this still physiological functioning path allows the action selection system to correct miscalculations. This could also explain why there is still slow but stable syllable sequencing (Table 2B, dark green) at very low GPi activity level (i.e., at low *wp*-values), where there are “only” oscillations and irregularities (Table 1B, light green and yellow) at very low levels of STN activity (i.e., at low *wt*-values). This interpretation is in agreement with the findings of Meissner et al. (2005), who found that DBS of STN has more negative effects on speech. We were not able to stabilize syllable sequences with low STN values, and instead saw only oscillatory behavior. With low levels of GPi activity, there were slow but stable syllable sequences, as were found in Tan et al. (2016), suggesting that reduction of GPi activity is more effective in recovering verbal fluency.

Nevertheless there are certain limitations of this model. It focuses on the effects of dopamine on striatal D1 and D2 receptors. However, dopamine also affects other nuclei in the basal ganglia. We have not considered these effects in the current approach. Also the model is complex and does not allow isolation of a single network mechanisms behind these observations. We show that inhibition of GPi and STN alleviate the impaired sequence generation and suggest this as an explanation of why DBS shows improvement in such cases. However, DBS is a complex stimulation, the effects of which could be multifaceted. The details of effect of DBS stimulation on the nuclei is beyond the scope of this work. Further, decreased activity in the STN and GPi does not arise as a result of recurrent connections and e.g., a given external input, but is set at input values (wt and wp).

As the mechanisms underlying DBS are still unclear (McIntyre et al., 2004; Johnson et al., 2008), our findings underline theories of DBS positing that the nuclei exposed to DBS are inhibited (Welter et al., 2004; Meissner et al., 2005). Future computer simulation studies may identify other worthwhile targets to influence or investigate other possible DBS mechanisms.

## Author contributions

VS developed the theory and designed the experiment, executed the simulations and wrote the manuscript. TS, TB, CE, and BK contributed to implementing the simulation in NENGO and PYTHON and editing the manuscript. BK conducted the experiments and wrote basic parts of the source code defining the neural model using NENGO and PYTHON.

### Conflict of interest statement

The authors declare that the research was conducted in the absence of any commercial or financial relationships that could be construed as a potential conflict of interest.
